# John C. Dalton, M. D., LL.D.

**Published:** 1889-03

**Authors:** 


John C. Dalton, M. D., LL. D., President of the College ot Physicians and Surgeons, died at his home in New York, February 12, 1889, at the age of sixty-four. To the older physicians residing in or near Buffalo, who remember this brilliant teacher with affectionate regard, the announcement of Dr. Dalton's death will be especially painful. It was in this city, as professor of physiology in Buffalo Medical College, that, fresh from the teachings of the great Bernard, he inaugurated the system of teaching and illustrating his subject by vivisections in the presence of his students. This had never yet been done in this country, and served to bring him a fame which justly and stoutly clung to his name through all his after-life.

Dr. Dalton's renown as scholar, teacher, and author, must endure so long as medicine has a literature or a student, and it will always be a pleasant thought to this Journal that he was one of its early contributors.

